# Linking the Tuneability and Defouling of Electrically Conductive Polyaniline/Exfoliated Graphite Composite Membranes

**DOI:** 10.3390/membranes11080631

**Published:** 2021-08-17

**Authors:** Lili Xu, Kunpeng Wang, Jun Wang, Darrell Alec Patterson

**Affiliations:** 1 State Key Laboratory of Environmental Aquatic Chemistry, Research Centre for Eco-Environmental Sciences, Chinese Academy of Sciences, Beijing 100085, China; junwang@rcees.ac.cn; 2 Department of Chemical Engineering, University of Bath, Bath BA2 7AY, UK; d.patterson@bath.ac.uk; 3 State Key Joint Laboratory of Environment Simulation and Pollution Control, School of Environment, Tsinghua University, Beijing 100084, China; wkp19@mails.tsinghua.edu.cn

**Keywords:** stimuli-responsive membranes, polyaniline, exfoliated graphite, electrically tuneable, fouling

## Abstract

Stimuli responsive membranes, which are able to respond to environmental stimuli, are attracting ever-increasing interests. In this study, we blended exfoliated graphite (EG) into the polyaniline (PANI) and developed PANI/EG composite membranes. The properties of the new generated membranes, especially the stimuli response properties (e.g., electrical tuneability, deformation), were studied. The fouling removal ability of the membrane under applied electrical potential was also investigated by using bovine serum albumin (BSA) as a model foulant. A flat membrane with defect-free surface and good adhesion to the support layer was formed by non-solvent induced phase separation method. The electrical conductivity of the formed PANI/EG composite membrane was (5.10 ± 0.27) ×10^−4^ S cm^−1^. The dynamic droplet penetration rate through the membranes showed an increase under applied electrical potential, which gives a preliminary quantitative indication of the electrical tuneability of the membranes. The membrane deformation appeared at a fast response under applied potential and recovered to its original position immediately when removing the applied potential. The application of electrical potential led to the removal of BSA foulant from the membrane surface as indicated by the increase in permeance of the fouled membrane on cleaning with 46.2% flux recovery ratio and increased BSA concentration in the wash solution. The electrically conductive PANI/EG composite membranes are able to respond to electrical stimuli, enabling a new range of potential applications including externally tuneability and in situ removal and control of fouling.

## 1. Introduction

A membrane is a selective barrier regulating the transport of substances from a process stream containing a mixture of components. The membrane can be used in a wide range of processes—from pharmaceutical manufacturing to wastewater treatment—to affect a selective separation, creating two purer process streams from a process stream containing a mixture of components. Typically, the physical and chemical properties of commercially available membranes are fixed during or after fabrication, and the membranes are relatively insensitive to response to the external stimuli [1,2]. Thus, the performance of membranes can be weakened by membrane fouling, since foulants deposited on the pore surface can reduce the pore size and hinder the interactions between the membrane and feeds [1,3]. Unalterable membrane properties and unavoidable membrane fouling may restrict the wide and efficient applications of conventional membranes in extended fields [4,5,6]. In this regard, there is a need to develop stimuli responsive membranes with tuneable properties and give a promising solution to in situ fouling removal using external stimuli [3,7].

Stimuli responsive membranes, otherwise called “smart”/“switchable”/“intelligent” /“adaptive” membranes, have been created by incorporating stimuli-responsive materials into membrane substrates as functional gates [1,7,8]. In response to external stimuli (e.g., pH, temperature, light, magnetic field, etc.), the functional gates provide a conformational switch for adjusting the membrane structure (pore size) and/or properties (mass transfer) and thus tuning the membrane performance [1,7]. Stimuli responsive membranes combine the advantages of conventional membranes and smart gates for enhanced membrane performance and applications [1]. For example, in terms of membrane fouling, if the pore size or morphology of the membranes can be enlarged by external stimuli, thus the fouling layer can be pushed through the membranes. Meanwhile, if the membrane surface properties can be changed by tuning the hydrophilicity of membranes and thus changing the affinity between foulants and membrane surface, resulting in fouling mitigation in situ. Based on this, the development of stimuli responsive membranes for in situ fouling removal will be a breakthrough in membrane science, and this forms the main motivation of this work [9,10,11].

Polyaniline (PANI), as an intrinsically conducting polymer, has been widely researched due to its conductive properties achieved by the protonation of imine nitrogens (=N–). It is particularly attractive for membrane separation, since its porosity can be controlled at a molecular level through simple acid/base doping/dedoping [12,13,14]. The transport properties of conducting PANI membranes, such as permeance and selectivity, could be tuned in situ by applying an electrical potential across small acid doped PANI membranes [15]. It was proposed that high potential can cause the movement of acid dopants, changing the dopants attachment or steric position in the polymer structure that would slightly swell the polymer chains, allowing permeance and selectivity to be tuned. However, small acid leaching during filtration led to weakened membrane tuneability and limited the further utilisation of PANI membrane [15]. To overcome the challenge, polymer acids (PAs) have been used as dopants to improve the acid stability. Our research group has recently showed that polymeric acids, poly(2-acrylamido-2-methyl-1-propanesulfonic acid) (PAMPSA), can produced stable membrane, possibly due to steric effects and the strong interaction between the acidic group of the polymeric acids and the PANI [16]. However, the electrical conductivity of the resulting PAMPSA doped PANI membrane was reduced to a low level, resulting in a limited tuneability under applied potential [16].

Exfoliated graphite (EG), a certain chemical or physical modification of the neat graphite, is well known to be a laminated and porous material with high electrical conductivity. It can serve as conductive bridges connecting PANI conducting domains and increasing the charge transfer mobility, resulting in an enhanced conductivity [17,18,19]. Incorporating conducting polymers like PANI into the interlayer spacing of EG has attracted considerable attention as a result of the high electrical conductivity [18,20]. Numerous potential applications have focused on supercapacitors [21], sensors [22], rechargeable batteries [23], conductive inks [24], microbial fuel cells [25], etc. To the best of our knowledge, there have been no reports of incorporating EG into PAs doped PANI membranes to improve the membrane conductivity, and then realise the tuneability under applied potential. Therefore, studies of combining EG with PAs doped PANI membrane will be investigated. A secondary dopant, applied to a primary-doped PANI, can induce dramatic conformation change in the molecular network of PANI, resulting in an increased electrical conductivity [26]. A long chain acid namely dodecylbenzene sulfonic acid (DBSA) possesses some attractive advantages like strong acidity, effective doping and plasticisation for PANI, and thereby can be considered as a promising secondary dopant to enhance the membrane conductivity [27,28]. Based on this, DBSA will be incorporated into the membrane to further improve the electrical performance of the membrane.

In terms of this, the main objective of this paper is to develop stable conducting PANI composite membranes which can exhibit stimuli response properties (e.g., electrical tuneability, deformation) under applied electrical potential. The primary application of the new generated membrane is to allow the fouling layer to be pushed off/through membrane by external potential. The mechanism of membrane tuneability and fouling removal behaviour under applied potential was also explored.

## 2. Materials and Methods

### 2.1. Materials

Acetone (≥99.8%), poly (2-acrylamido-2-methyl-1-propanesulfonic acid)/PAMPSA (MW = 800,000 g mol^−1^, 10 wt% in water), fluorescein isothiocyanate (FITC, ≥90.0%), hydrogen peroxide (H_2_O_2_, 30 wt% solution in water) and sulphuric acid (H_2_SO_4_, 96% solution in water) were purchased from Fisher. Aniline (≥99.5%, Heysham, UK), ammonium persulfate (APS, ≥99.0%), hydrochloric acid (HCl, 37% solution in water), isopropyl alcohol (≥99.0%), N-methyl-2-pyrrolidone (NMP, ≥99.0%), 4-methyl piperidine (4-MP, 96.0%) and BSA (≥98.0%) were purchased from Sigma-Aldrich, Dorset, UK. Graphite powder (2–15 μm, 99.9995% (metals basis)) was purchased from Alfa Aesar, Heysham, UK.

### 2.2. Fabrication of PANI/EG Composite Membranes

#### 2.2.1. Synthesis of PANI-PAMPSA Complex

The synthesis of PANI-PAMPSA complex was followed by a recently developed method [16]. Aniline (0.06 mol), at 4:1 monomer to PAMPSA repeat unit molar ratio, was dissolved in the acid solution (0.1 M). The oxidising agent APS (1:1 APS to aniline monomer molar ratio) dissolved in DI water was slowly added into the mixture of aniline and PAMPSA. The reaction temperature was set at 15 °C with reaction time of 24 h. The reactant product was washed with DI water and acetone, and then dried at 60 °C for 24 h. After that the obtained PANI-PAMPSA complex was ground to obtain a fine product with black green colour. Figure S1 (supplementary material) displays the chemical oxidation process used for the synthesis of PANI-PAMPSA complex.

#### 2.2.2. Preparation of Exfoliated Graphite

EG was synthesised using hydrogen peroxide–sulphuric acid methods, following the process reported in the literature [17,29]. The graphite powder (20 g) was added in the concentrated H_2_SO_4_ (200 mL) and stirred until well dispersed, and then H_2_O_2_ (20 mL) was added into the mixture followed by stirring for 2 h. The as-treated graphite was washed by DI water until the pH level of the filtrate reached 6 and then dried at 100 °C for 24 h. The dried powder was heated in a furnace at 700 °C for 2 min. The obtained powder was dispersed in a 50 wt% isopropyl alcohol solution and sonicated for 2 h, and then washed with DI water and dried under vacuum at 60 °C for 24 h.

#### 2.2.3. Membrane Fabrication

The membrane was fabricated via the non-solvent induced phase separation (NIPS) method using water as a non-solvent. EG (5.78 g) was added to the mixture of NMP (20.64 mL) and 4-MP (2.35 mL) with the aid of ultrasonication (180 W, 50 KHz) at a low temperature (5 °C) for 2 h to allow good dispersion, then the previously synthesised PANI-PAMPSA complex (5.78 g) was slowly added into the mixture in portions by overhead stirring and ultrasonicated at a low temperature to form a homogeneous mixture. The membrane was casted at 250 µm using an adjustable doctor blade onto a polyethylene/polypropylene (PE/PP) backing layer (Novatexx 2431, Freudenberg Filter, Crewe, UK). After evaporating for 15 s in the air, the cast membrane was immersed in DI water at a room temperature. The coagulation water was changed several times to remove any residual solvent. For the secondary doping, the obtained membrane was immersed in DBSA solution (0.1 M) at 80 °C for 6 h, and then rinsed with DI water for later use. Figure S2 (supplementary material) displays the membrane preparation process.

### 2.3. Characterisation Techniques

Attenuated total reflectance Fourier transform infrared spectroscopy (ATR-FTIR; PerkinElmer, Billerica, MA, USA) was used to analyse the functional groups of samples. The membrane conductivity was measured using a four-point probe conductivity meter (RM3000, JANDEL) in a sheet resistance mode. The morphologies of the samples were imaged by scanning electron microscopy (SEM; JSM-6480 LV, JEOL, Oberkochen, Germany). The cross-sections of the membrane were prepared by fracturing in liquid nitrogen. The membrane samples were sputter coated with gold before being imaged with SEM. The acceleration voltage was 3 kV and 10 kV for graphite samples and membrane samples, respectively. Red, green and blue (RGB) were used to give a quantitative evaluation of the colour of the membrane. The samples were scanned by a digital scanner (CanoScan 9000F, Canon, Tokyo, Japan) and Image J software was used to calculate the RGB value. The detailed calculation of RGB has been previously described [15].

### 2.4. Membrane Transport Property

Membrane filtration was carried out in a dead-end filtration cell (HP 4750, Sterlitech, Kent, Washington, DC, USA). Membrane pieces with an effective filtration area of 14.6 cm^2^ were cut and placed in a stainless metal plate. Nitrogen gas was used as the driving force, and a stirrer was used to minimize the concentration polarization at a stirring rate of 300 rpm. The membrane was pre-conditioned with DI water until a steady flux was achieved. Methods for determining flux and rejection using DI water or polyethylene glycols (PEGs) mixtures (of PEG 1000, 1500, 2000, 3000, 4000 and 6000) have been described in the previous study [30]. Permeate from the filtration was collected and recorded by a digital mass balance. High-performance liquid chromatography (HPLC) coupled with an evaporative light scattering detector (ELSD) were used for the identification of individual PEG oligomers in the feed, permeate and retentate [30].

### 2.5. Membrane Electrical Tuneability Analysis

#### 2.5.1. Dynamic Droplet Penetration Analysis

The dynamic droplet penetration through the membranes with or without the applied potential was measured by a contact angle goniometer (OCA 15Pro, Dataphysics, Filderstadt, Germany). Electrodes were connected to an analytical platform to provide electrical potential across the membrane (Figure S3 in the supplementary material). A droplet of water (2.0 µL) was dropped onto the membrane surface and a video camera was used to record images of the droplet every 1 s until 12 s with and without applied potential. The programme software was used to calculate the effective contact angle and droplet height to analysis the penetration of droplet through the membrane.

#### 2.5.2. Membrane Deformation Analysis

Membrane deformation was analysed by using the aforementioned contact angle goniometer. The membrane rinsed with DI water was blotted in paper to remove excessive water. The images of wet membranes under different applied potentials were taken, and the distance between the membrane surface and baseline was calculated. The difference of the distance indicated the membrane deformation extent under applied potential.

### 2.6. Membrane Defouling Analysis

#### 2.6.1. Membrane Fouling Experiment

Membranes were examined for their potential for in situ fouling removal by BSA foulant. The defouling experiment was performed under an applied potential at a room temperature using a static rig as shown in Figure S4 in the supplementary material. Firstly, DI water was used to measure the flux of virgin membranes, and then 1.0 g L^−1^ BSA solution (200 mL) was used to foul the membrane, after that DI water was run again to determine the flux of the fouled membranes. All the filtration experiments were operated in a dead-end filtration mode.

#### 2.6.2. Membrane Defouling Experiment

The fouled membranes were firstly immersed in 800 mL water (as termed wash solution) and then an electrical potential (30 V) was applied on the membrane for 120 min. The samples of the wash solution were taken every 30 min (e.g., 0, 30, 60, 90 and 120 min). Membranes following this treatment are termed cleaned. DI water was used to measure the flux of the cleaned membranes in a dead-end filtration mode. In addition, control experiments were run on fouled membranes without applied potential and unfouled membranes with applied potential.

#### 2.6.3. Defouling Analysis

The components of the wash solution were analysed by UV-Vis to evaluate membrane defouling performance under applied potential. SEM (JSM-6480LV, JEOL, Freising, Germany) and confocal scanning laser microscopy (CSLM) (Carl Zeiss LSM, Oberkochen, Germany) were performed to determine the surface difference among virgin, fouled and cleaned membranes and distinguish the membrane defouling behaviour by the application of external potential. The acceleration voltage of SEM was set to 10 kV for the samples. The membrane samples were stained using FITC dye for 1 h and then washed with phosphate-buffered saline to remove the excessive dye, after that the membrane samples were viewed by using CSLM.

## 3. Results

### 3.1. Chemical Properties, Morphology and Performance Characterisation of Membranes

A flat membrane with a defect-free surface and good adhesion to the support layer was prepared under the casting conditions considered. The fabricated membrane displayed a greenish dark colour as a result of EG incorporation and acid doping (Figure S5 in the supplementary material), similar to the reported colour concerning the PANI composite membrane with carbon nanomaterials [31]. The chemical structure, electrical properties, morphology and transport properties of the new generated PANI/EG composite membrane were analysed below.

#### 3.1.1. FTIR and Electrical Conductivity of PANI/EG Composite Membranes

**Figure 1:**
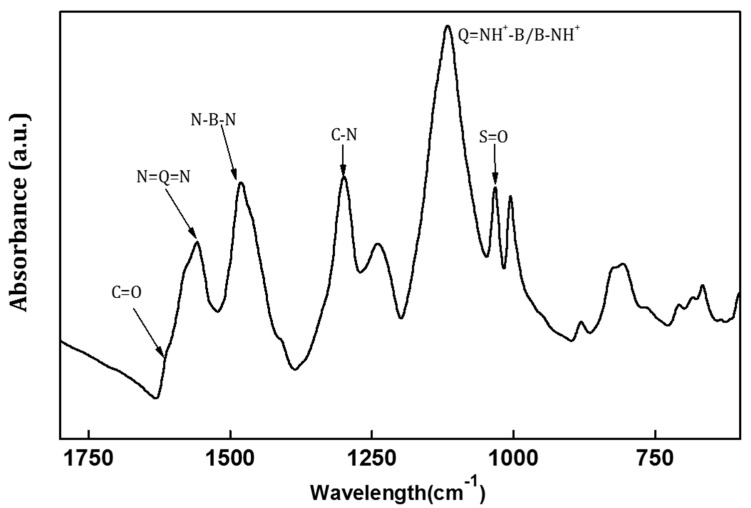
FTIR spectra of the PANI/EG composite membrane.

Figure 1 indicates the characteristic peaks present in the PANI/EG composite membrane. The characteristic bands observed at 1559 cm^−1^ and 1481 cm^−1^ correspond to the absorption peaks of the quinoid (N=Q=N) and benzenoid (N–B–N) rings, respectively. Generally, the characteristic bands of quinoid and benzenoid rings appeared at approximately 1598 cm^−1^ and 1498 cm^−1^ in undoped PANI membrane [15] (Figure S6 in the supplementary material). The quinoid stretching mode red shifted from 1598 cm^−1^ to 1559 cm^−1^, implying the interaction between π-conjugated structure and imine nitrogens of PANI, transforming the imine groups (=N–) into amine groups (–N–) [32]. The band at 1295 cm^−1^ corresponds to C–N stretching vibrations of secondary amine of PANI backbone. The peak at 1033 cm^−1^ was assigned to S=O stretching of sulfonic acid group, and the band at 1653 cm^−1^ corresponded to C=O stretching of PAMPSA, which was indicative of the incorporation of acids into membranes [33,34,35,36]. The band at 1120 cm^−1^ can be assigned to the plane bending vibration of a charged structure (Q=NH^+^–B or B–NH^+^) formed during protonation, associated with the degree of charge delocalisation on the polymer backbone [37].

The electrical conductivity of the PANI/EG composite membrane was (5.1 ± 0.3) × 10^−4^ S cm^−1^. Compared to the membrane previously prepared in the lab, the conductivity was improved by two orders of magnitude due to the addition of EG and DBSA [16]. The reason was that EG can serve as conductive bridges connecting PANI conducting domains and promoted more activated electrons, which enabled us to enhance the electron transfer and charge mobility through the polymer structure [38]. Besides, secondary DBSA doping induced structural relaxations of PAMPSA doped PANI and promoted more efficient charge transfer, contributing to an improvement in the conductivity of PANI composites. The electron transfer between secondary doped PANI and EG became less restricted compared to primary PAMPSA doped PANI with EG. Therefore, it could lead to an improvement in both the carrier number and charge mobility, contributing to an overall enhancement of electrical conductivity.

#### 3.1.2. Morphology of EG and PANI/EG Composite Membranes

**Figure 2:**
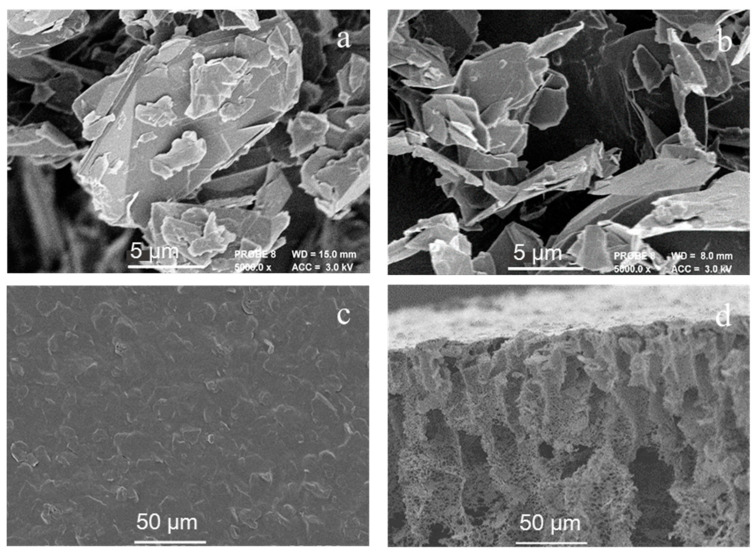
Microstructure of graphite (**a**) and exfoliated graphite (**b**), morphologies of surface (**c**) and cross-section (**d**) of the PANI/EG composite membrane.

Figure 2 shows the microstructure of graphite and exfoliated graphite, the surface morphology and cross-section of the PANI/EG composite membrane. As shown in Figure 2a,b, the graphite was more clustered due to the layered nature while the exfoliating treatment of the graphite resulted in better delamination of the graphite layers. Such a laminated morphology is desirable, as it can provide interlayer spacing for the formation of conductive networks in the polymer matrix [39].

The surface morphology of the membrane in Figure 2c showed that EG nanosheets were uniformly dispersed in the matrix and a good interfacial contact was established at the interface, forming a conducting network. The cross-section of the membrane consisted of a thin top layer, a transition region and a porous layer with large macroscopic-voids, which indicates rapid demixing during the non-solvent induced phase inversion process.

#### 3.1.3. Transport Properties and Filtration Stability of PANI/EG Composite Membranes

**Figure 3:**
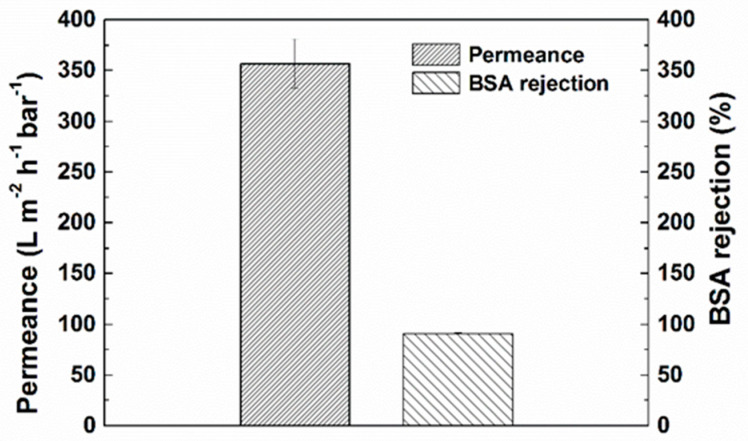
Permeance and BSA rejection of the PANI/EG composite membrane. Experimental conditions: operating pressure 2 bar, [BSA] = 1.0 g L^−1^, room temperature.

Water permeance of PANI/EG composite membranes was conducted in dead-end filtration and BSA nanoparticles were utilised to evaluate the membrane rejection. Figure 3 illustrates the permeance and BSA rejection of the membrane. The membrane permeance was 357 ± 24 L·m^−2^·h^−1^·bar^−1^ and the membrane exhibit above 90% rejection of BSA (MW = 66,000 g mol^−1^), which suggested that the MWCO of membranes was less than 66,000 g mol^−1^ with BSA as the MWCO probe.

**Figure 4:**
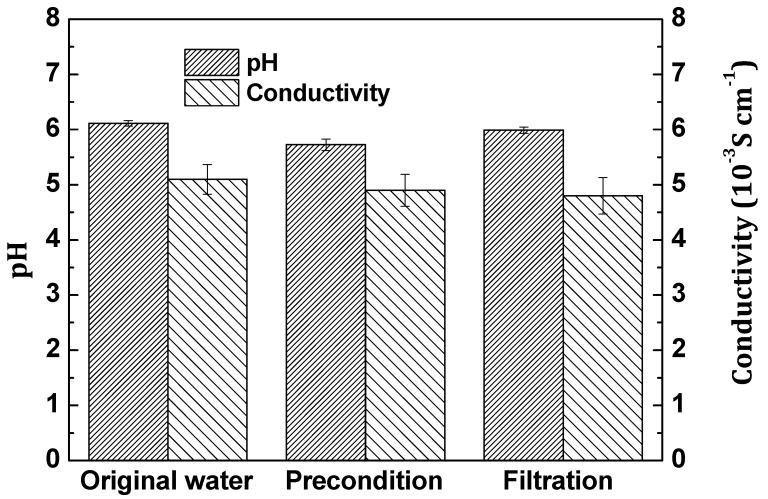
The membrane conductivity and pHs of the permeate (before filtration, after preconditioning and after the actual filtration) in the dead-end filtration using water as feed. Experimental conditions: operating pressure 2 bar, room temperature.

In order to test the membrane stability during filtration, the membrane conductivity and pHs of the membrane permeate during different filtration stages were measured. Figure 4 presents the membrane conductivity and pHs of the permeate (before filtration, after preconditioning and after the actual filtration) in the dead-end filtration using water as feed. In the different filtration stages, the membrane conductivity and pHs of the membrane permeate are kept stable, which indicated that the PANI/EG composite membranes were stable in filtration.

EG with high specific surface area facilitated the intercalation of PANI-PAMPSA into its nanosheets in the solution mixing process, and so was able to quickly reach an equilibrium state [40,41]. Some studies have shown that PANI-PAMPSA can function as an acceptor compound via the radical cations generated by PAMPSA, and graphite can act as the electron donor [20]. The strong interaction between PANI and the basal plane of EG surfaces led to charge stabilisation and an increased tendency for PANI-PAMPSA to coat on the EG to form a stable composite [42]. Additionally, DBSA with long alkyl side chains had weak molecular mobility compared to small acids, and the bonding between imine nitrogen of PANI and sulfonic acid group of DBSA immobilised DBSA in the chain structure. These all made it more difficult for leaching to happen [43].

### 3.2. Electrically Stimuli Response of PANI/EG Composite Membranes

#### 3.2.1. Initial Tuneability Assessment of PANI/EG Composite Membranes

**Figure 5:**
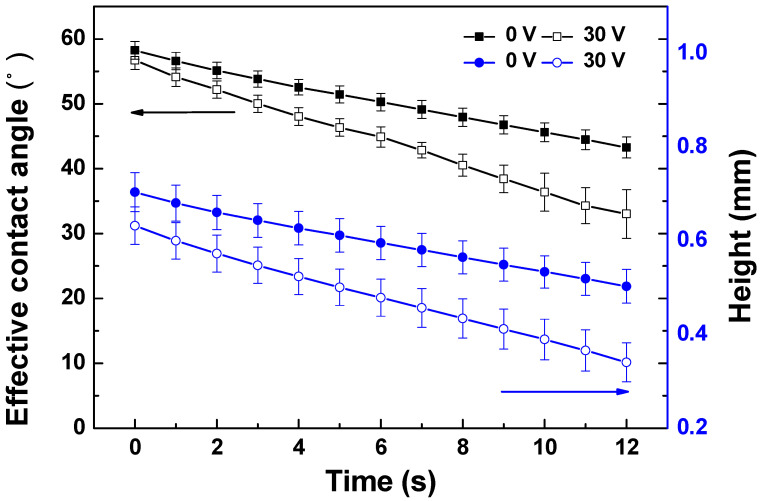
The effective contact angle and droplet height change of PANI/EG composite membranes over time with and without applied potential (30 V).

The measurement of dynamic contact angle change with or without applied potential can be used to study the electrically tuneable permeation of water through the membrane, which suggested the electrically changeable/tuneable permeation behaviour of the conducting PANI membranes [15]. The variation of effective contact angle and droplet height with time indicated the water permeation properties under gravity. Figure 5 shows the change of effective contact angle and droplet height over time with and without applied potential. As can be seen, the applied potential had a significant impact on the water permeation rate through the membranes. The rate of effective contact angle varied from 1.3 to 2.0 θ s^−1^ and the rate of droplet height changed from 1.7 × 10^−2^ to 2.4 × 10^−2^ mm s^−1^ with and without applied potential. This gives a preliminary quantitative indication of the electrical tuneability of the membranes, strongly indicating that the permeation rate of conductive PANI/EG composite membranes can be tuned by applied electrical potential.

Water is neutrally charged, and thus no charge repulsion or attraction forces are expected to affect its permeability. Applying an electrical potential to the membrane resulted in a transition to a higher water permeation rate, indicating the bulk volume swelling is likely to be the main reason for the increased permeation rate [44]. EG possesses highly mobile electrons, and polarons were formed to move along the polymer backbone by applying an external potential, causing the movement of counterions in the polymer chains. This resulted in the reorganization of the polymer structure, permitting a more open membrane structure accompanied by the expansion of membrane [45]. The created voids or free volume allowed more water to pass through the membrane [46]. It was interesting to find that the membrane exhibited obvious deformation under applied potential (in Section 3.2.2), which has not been found in the PANI membrane with low electrically conductivity [16]. This could further confirm the volumetric deformation of PANI/EG composite membranes by the application of external potential.

#### 3.2.2. Deformation of PANI/EG Composite Membranes under Applied Potential

**Figure 6:**
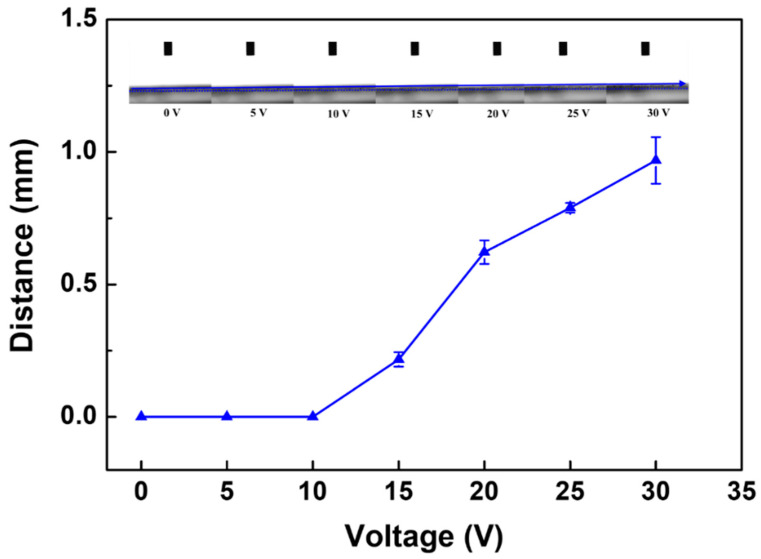
The deformation of PANI/EG composite membranes in response to the applied potential.

Figure 6 shows the membrane deformation degree under different applied potential. It was found that the deformation of PANI/EG composite membranes appeared at a fast response under applied potential and recovered to its original position almost immediately when removing applied potential. The main reason for the deformation is likely due to the applied voltage providing extra charge to the already positively charged membrane surface, which promotes the movement of counterions in the membrane structure. The movement of the counterions increases the steric hindrance between the chains and thus compel the chains to loosen or the pores to open. Additionally, the fast response is likely due to the rapid excitation of electrons by the applied voltage, which also confirmed the preliminary design that the applied potential was used to stimulate the tuneablity instead of pH or temperature to alleviate time lag. It was also found that the membrane deformation was highly applied potential dependent. The deformation degree increased with an increase in the applied potential from 0 to 30 V. The higher applied potential provided more electrons and accelerated the charge delocalisation, allowing more significant polymer chain movement, and thus promoted more significant membrane deformation. A series of photos demonstrating the deformation of membranes in response to the applied potential are shown in Figure S7 in the supplementary material.

### 3.3. Defouling of PANI/EG Composite Membranes under Applied Potential

#### 3.3.1. Membrane Defouling Performance in Filtration

**Figure 7:**
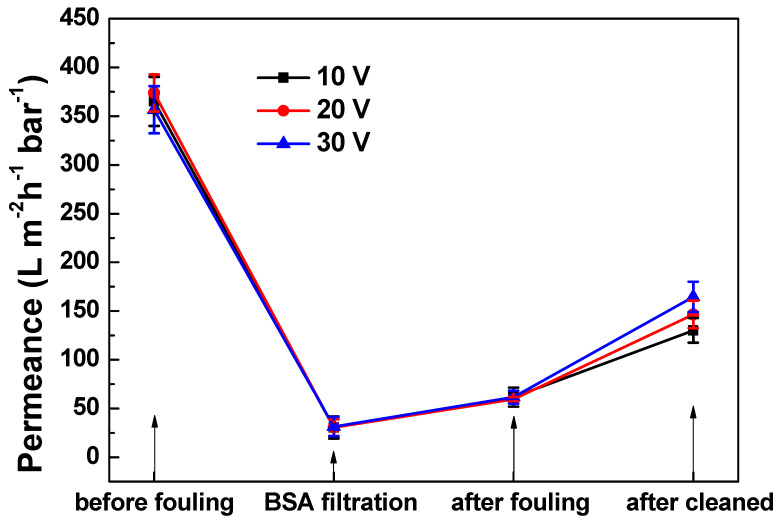
Permeance of PANI/EG composite membranes (virgin, BSA fouled, after fouled and cleaned) at different applied potentials. Experimental conditions: applied potential 10–30 V, operating pressure 2 bar, [BSA] = 1.0 g L^−1^, room temperature.

**Figure 8:**
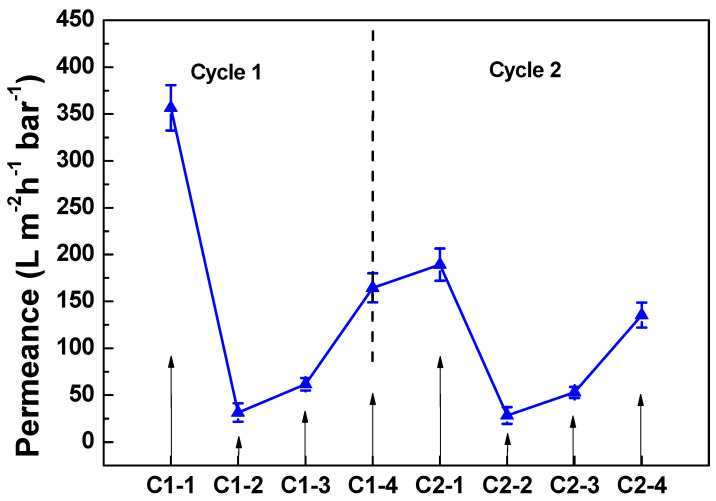
Permeance of PANI/EG composite membranes (virgin, BSA fouled, after fouled and cleaned) under the applied potential of 30 V at two cycles (C1-1 = virgin (Cycle 1), C1-2 = BSA fouled (Cycle 1), C1-3 = after fouled (Cycle 1) and C1-4 = cleaned (Cycle 1); C2-1 = virgin (Cycle 2), C2-2 = BSA fouled (Cycle 2), C2-3 = after fouled (Cycle 2) and C2-4 = cleaned (Cycle 2)). Experimental conditions: applied potential 30 V, operating pressure 2 bar, [BSA] = 1.0 g L^−1^, room temperature.

The permeance of virgin, fouled and cleaned PANI/EG composite membranes is shown in Figure 7. There was a significant permeance reduction after BSA fouling—from 357 ± 24 to 32 ± 10 L·m^−2^·h^−1^·bar^−1^. Observations of membranes after fouling showed a gel-like BSA fouling on the surface of the membrane, which was, therefore, the major reason for the permeance reduction. The presence of applied potential promoted the removal of BSA fouling from PANI/EG composite membrane, and the membrane defouling property has a positive relationship with the applied potential. The permeance increased to approximately three times that of the fouled membrane, and the membrane permeance recovery arrived at 46.2% of the virgin membrane under the applied potential of 30 V. This result is comparable to the published work, which showed that applied potential can lead to 43% reduction of the operating pressure [10]. Besides, another cycle has been added to investigate the membrane durability, which stands for long-term operation and stability of membranes. Figure 8 shows the membrane permeance under the applied potential of 30 V at different cycles. It was found that the membranes exhibit good fouling reduction behaviour at two cycles, indicating that PANI/EG composite membrane has the potential for a long period operation.

BSA concentration in the wash solution was also measured to evaluate the foulants washed away from the membrane and to further confirm the fouling removal behaviour under applied potential. BSA concentration in the wash solution of PANI/EG composite membranes with time was displayed in Figure S7 (supplementary material). The membrane showed a significant fouling removal response in the first 90 min and the BSA concentration in the wash solution was steadily improved with time. There was no obvious increase was observed from 90 to 120 min and the final BSA concentration in the wash solution was (13.7 ± 0.5) × 10^−2^ g L^−1^, suggesting that most of the foulants were removed in the first 90 min.

#### 3.3.2. Characterisation of Membrane Fouling Reduction

**Figure 9:**
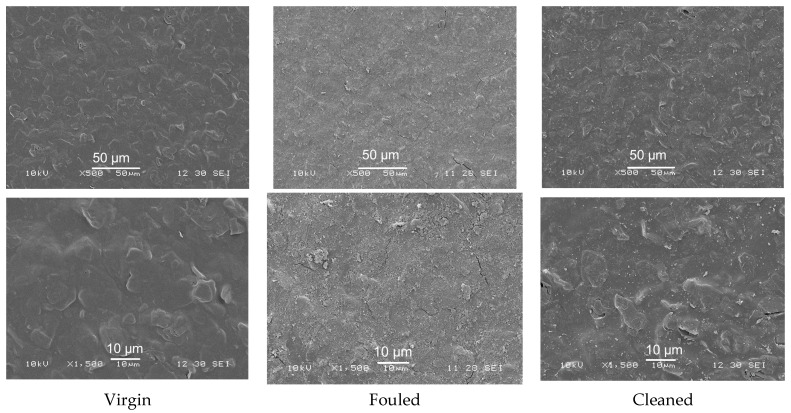
SEM images of virgin, BSA fouled and cleaned (**left** to **right**) PANI/EG composite membranes (applied potential = 30 V) with scale bar of 50, 10 µm (**top** to **bottom**), respectively.

**Figure 10:**
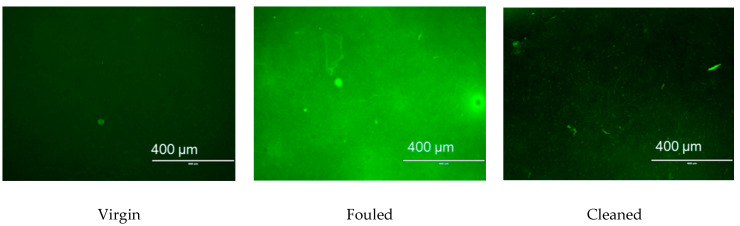
CSLM images of virgin, BSA fouled and cleaned (**left** to **right**) PANI/EG composite membranes (applied potentable 30. V) with scale bar of 400 µm.

Figure 9 presents the surface morphology of virgin, fouled and cleaned PANI/EG composite membranes (applied potential of 30 V). As can be seen, the membrane was fully covered by foulants after BSA filtration. The application of an electrical potential removed most of the contaminants on the membrane surface. Figure 10 shows the CSLM images of virgin, fouled and cleaned PANI/EG composite membranes. The membrane exhibited a dark green colour from the incorporation of fluorescein. The presence of BSA foulants on the membrane surface changed the colour into much greener one. After applying potential, most of the membrane surface changed back to dark green colour, suggesting that only few foulants were left on the membrane surface. The obtained CSLM images agree well with SEM images in Figure 9. It can be concluded that that most of the surface foulants on the membranes were removed by the application of electrical potential. This indicates that in pore (irreversible fouling) is the main fouling zone not removed by the applied potential treatment and should be the focus of future work.

**Figure 11:**
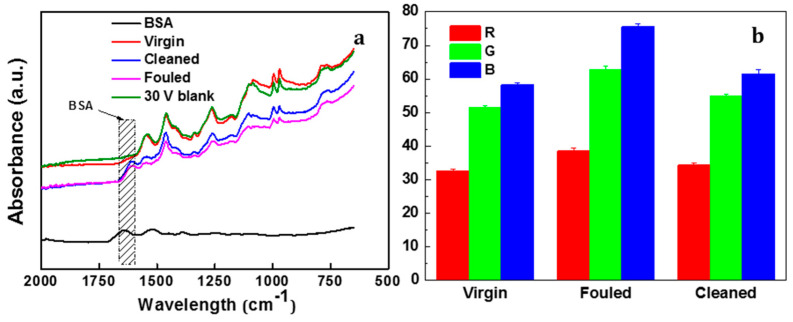
(**a**) FTIR and (**b**) colour change of PANI/EG composite membranes (virgin, BSA fouled and cleaned) (applied potential = 30 V).

Figure 11a presents the FTIR spectra of virgin, fouled and cleaned PANI/EG composite membranes. There are two regions in the BSA spectra, namely 1700–1600 cm^−1^ and 1550–1500 cm^−1^, unique to the protein secondary structure amide I and amide II, respectively. The amide I region is typically used for BSA structure analysis due to signal intensity [47]. Therefore, this region would be used for fouling study. Virgin membrane and a control test of 30 V without fouling displayed a similar spectrum, indicating that the membrane and polymer does not break down at the applied potential of 30 V. The amide I functional group of BSA was observed in the fouled membrane, again confirming that fouling occurred. After applying the external applied potential, the imide I peak still appeared in the cleaned membrane, suggesting that the electrical potential application can remove parts of the BSA foulant, but not all the foulants.

Colour analysis RGB (Red, green and blue) was used to quantitatively evaluate the colour change among the virgin, BSA fouled and cleaned of PANI/EG composite membranes. Figure 11b shows that the colour of fouled membrane almost recovered to the virgin state, indicating most of the contaminants were removed from the membrane surface. This agrees well with the previous results, suggesting that applied potential facilitated the in situ defouling behaviour of the PANI/EG composite membrane.

#### 3.3.3. Mechanisms of Membrane Fouling Reduction

The externally electrical potential triggered in situ fouling removal on the electrically conductive PANI/EG composite membrane and there were several major mechanisms contributed to the electricity driven fouling reduction [32,46,48].

On one hand, the applied potential could cause the movement of counterions in the polymer chains and trigger the reorganization of polymer structures, leading to the volumetric deformation and permitting a more opened membrane structure, which was also confirmed by membrane deformation phenomenon in Section 3.2.1. This could facilitate the fouling reduction in terms of pore plugging in the membrane.

On the other hand, the applied current across the conductive membrane provides a large number of free electrons, causing the oxidation and degradation of contaminants. The oxidation included direct oxidation driven by contact with conducting membranes and indirect oxidation by anodic production of an aqueous oxidant (e.g., hydrogen peroxide) [49]. The electrolytic oxidation can lead to the degradation or dehydration of foulants like protein, resulting in the release of deposited contaminants from membranes. BSA concentration in the wash solution of PANI/EG composite membranes with time was shown in Figure S8.

Additionally, the electrically conductive membrane serves as working electrodes by the application of electrical potential. The water can be electrolysed into hydrogen or oxygen molecules upon electrical potential. The generated gas bubbles at the interface of foulants and membranes can force the deposited BSA to detach from the solid–liquid interface and attach to the liquid–vapour interface. In this way, the protein at the liquid–vapour interface can be pushed away while the protein at the solid–liquid interface stays on the membrane surface [48,50]. These all contributed to the defouling behaviour of the conductive PANI/EG composite membrane under applied potential.

## 4. Conclusions

In this study, EG was incorporated into the PANI membrane by solution mixing, and a flat PANI/EG composite membrane with defect-free surface and good adhesion to the support layer was formed by NIPS method. The permeance of the new generated membrane was 357 ± 24 L·m^−2^·h^−1^·bar^−1^ and the membrane exhibit above 90% rejection of BSA. The electrical conductivity of the membrane was (5.1 ± 0.3) × 10^−4^ S cm^−1^. The applied potential had a significant impact on the dynamic droplet penetration rate through the membrane, with the rate of effective contact angle varying from 1.3 to 2.0 θ s^−1^, and the rate of droplet height changing from 1.7 × 10^−2^ to 2.4 × 10^−2^ mm s^−1^. This gives a preliminary quantitative indication of the electrical tuneability of the membranes. The membrane deformation appeared at a fast response under applied potential, the deformation was found to be highly applied potential dependent and the deformation degree increased with an increase in applied potential. Application of an electrical potential produced in situ fouling removal of BSA from the membrane, with flux recovered to 46.2% of the initial flux. Overall, these results suggest that the electrically conductive PANI/EG composite membranes have a great potential to respond to electrical stimuli, allowing in situ membrane fouling removal under applied potential. 

## Data Availability

Not applicable.

## Supplementary Materials

The following are available online at https://www.mdpi.com/article/10.3390/membranes11080631/s1. Figure S1: The chemical oxidation process used for the synthesis of PANI-PAMPSA complex. Figure S2: Figure S2. The preparation process of PANI/EG composite membrane. Figure S3: The electrically connected membrane assembly in the contact angle goniometer used for electrically connected dynamic droplet penetration analysis as an initial assessment of the electrical tuneable permeation properties of the PANI/EG composite membrane. Figure S4: Setup for evaluating membrane fouling removal. Figure S5: The appearance of the PANI/EG composite membrane produced by NIPS method. Figure S6: The characteristic bands of quinoid and benzenoid rings in the undoped PANI membrane. Figure S7: A series of photos demonstrating the deformation of the PANI/EG composite membrane in response to the applied potential. Figure S8: BSA concentration in the wash solution of PANI/EG composite membranes with time.
