# Supplementary material for: Linking the Tuneability and Defouling of Electrically Conductive Polyaniline/Exfoliated Graphite Composite Membranes
Source: Membranes (Basel). 2021 Aug 17;11(8):631. doi: 10.3390/membranes11080631 (PMC8400208; doi:10.3390/membranes11080631)
Supplement: Supplementary file 1 [file membranes-11-00631-s001.zip › membranes-1328624-supplementary.pdf]

membranes

Supplementary Material

Linking the Tuneability and Defouling of Electrically Conductive Polyaniline/Exfoliated Graphite Composite Membranes

Lili Xu [1,2,*], Kunpeng Wang [3], Jun Wang [1] and Darrell Alec Patterson [2]

1 State Key Laboratory of Environmental Aquatic Chemistry, Research Centre for Eco-Environmental Sciences, Chinese Academy of Sciences, Beijing 100085, China; junwang@rcees.ac.cn
2 Department of Chemical Engineering, University of Bath, Bath BA2 7AY, UK; d.patterson@bath.ac.uk
3 State Key Joint Laboratory of Environment Simulation and Pollution Control, School of Environment, Tsinghua University, Beijing 100084, China; wkp19@mails.tsinghua.edu.cn
* Correspondence: llxu@rcees.ac.cn

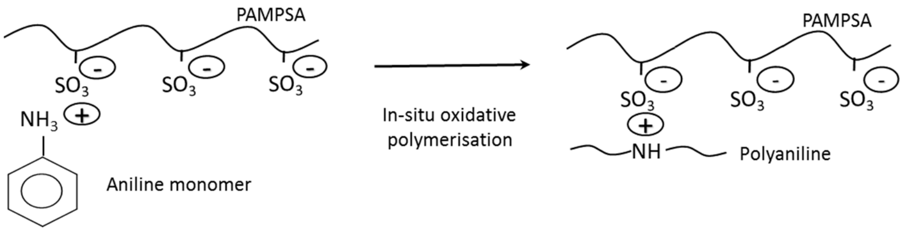

Figure S1. The chemical oxidation process used for the synthesis of PANI-PAMPSA complex.

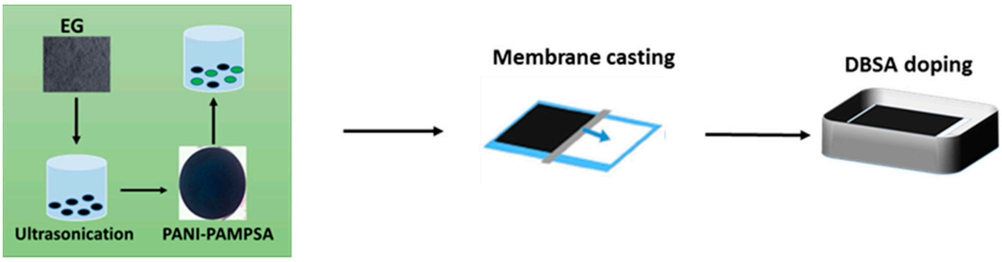

Figure S2. The preparation process of PANI/EG composite membrane.

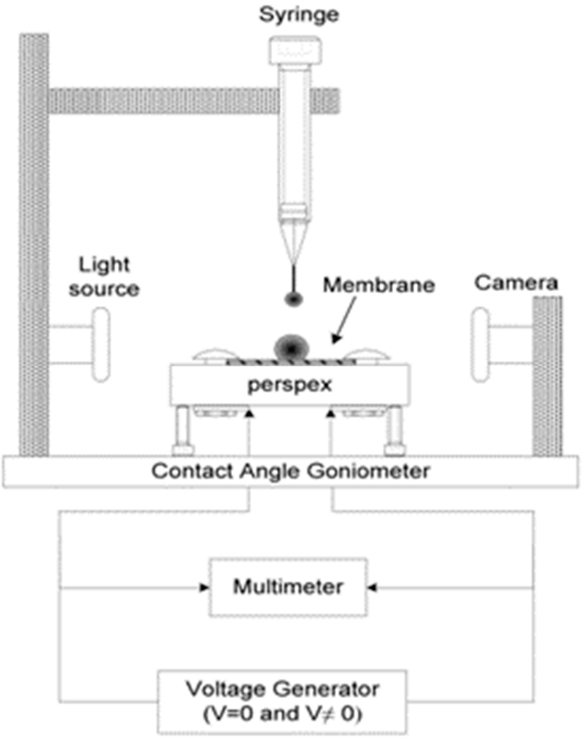

Figure S3. The electrically connected membrane assembly in the contact angle goniometer used for electrically connected dynamic droplet penetration analysis as an initial assessment of the electrical tuneable permeation properties of the PANI/EG composite membrane.

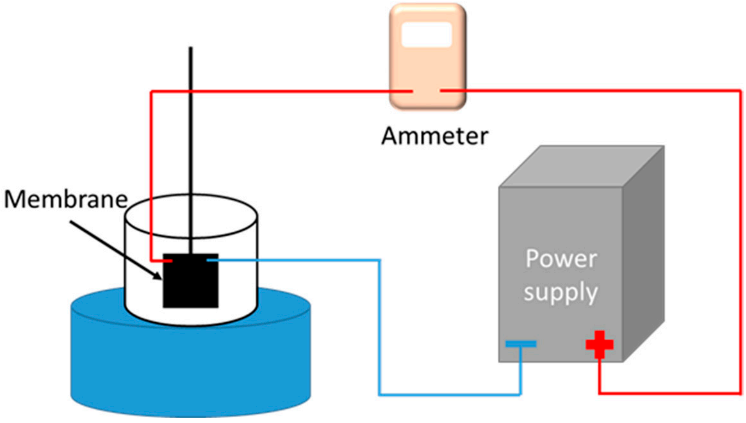

Figure S4. Setup for evaluating membrane fouling removal.

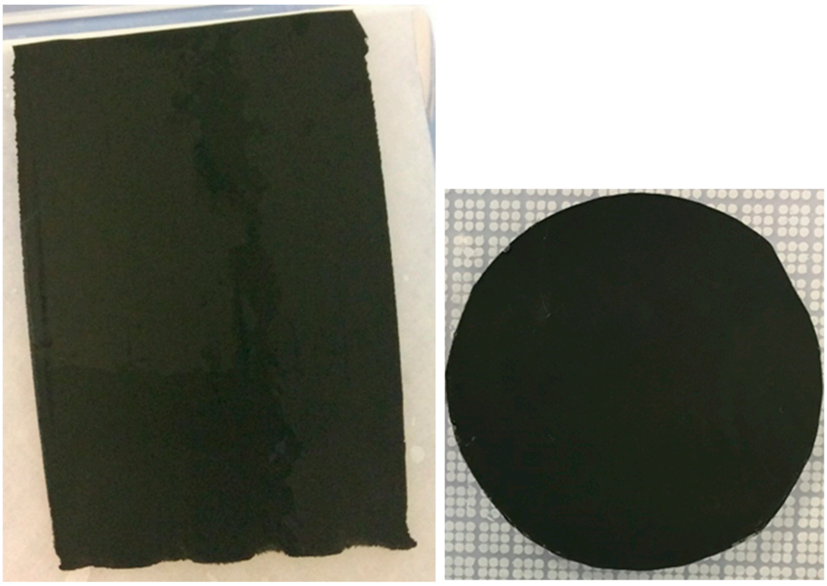

Figure S5. The appearance of the PANI/EG composite membrane produced by NIPS method.

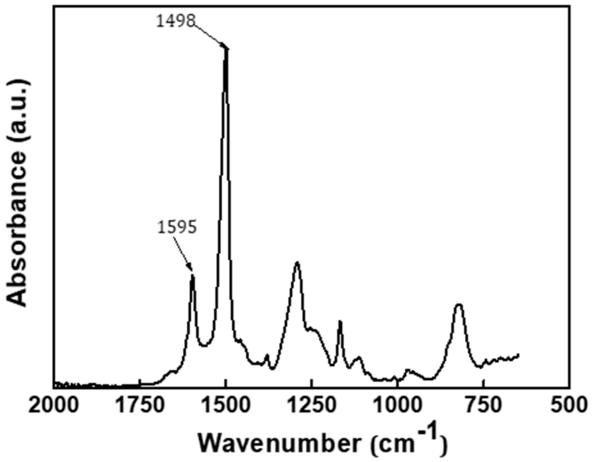

Figure S6. The characteristic bands of quinoid and benzenoid rings in the undoped PANI membrane.

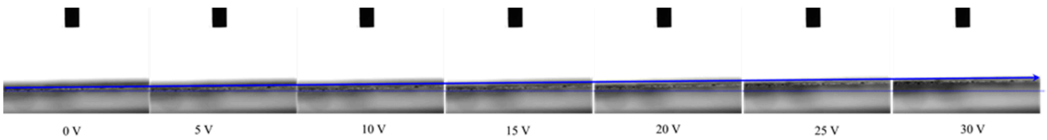

Figure S7. A series of photos demonstrating the deformation of the PANI/EG composite membrane in response to the applied potential.

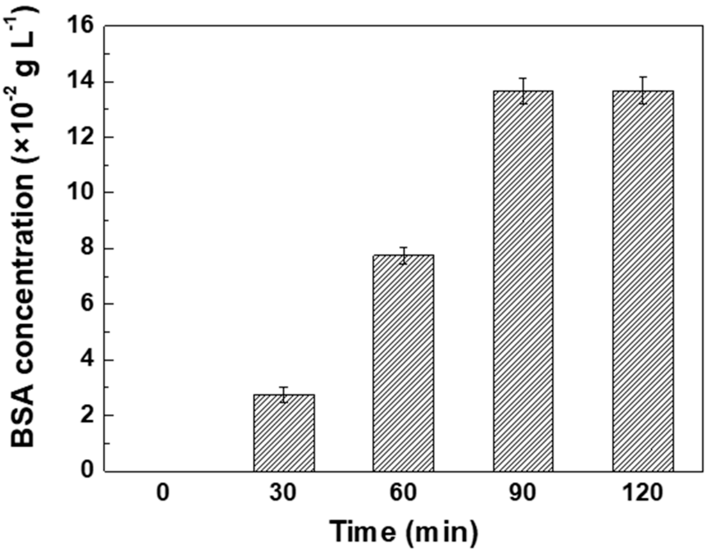

Figure S8. BSA concentration in the wash solution of PANI/EG composite membranes with time.
